# Phylogeography and Demographic History of *Babina pleuraden* (Anura, Ranidae) in Southwestern China

**DOI:** 10.1371/journal.pone.0034013

**Published:** 2012-03-20

**Authors:** Zejun Li, Guohua Yu, Dingqi Rao, Junxing Yang

**Affiliations:** 1 State Key Laboratory of Genetic Resources and Evolution, Kunming Institute of Zoology, the Chinese Academy of Sciences, Kunming, China; 2 Graduate University of the Chinese Academy of Sciences, Beijing, China; American Museum of Natural History, United States of America

## Abstract

Factors that determine genetic structure of species in southwestern China remain largely unknown. In this study, sequences of two mitochondrial genes (COI and cyt *b*) were determined to investigate the phylogeography and demography of *Babina pleuraden*, a pond frog endemic to southwestern China. A total of 262 individuals from 22 populations across the entire range of the species were collected. Our results indicate that *B. pleuraden* comprises five well-supported mitochondrial lineages roughly corresponding to five geographical areas. The phylogeographic structure of *B. pleuraden* has been shaped primarily by the unique regional responses of the Yunnan Plateau to the rapid uplift of the Qinghai-Tibetan Plateau occurred *c*. 2.5 Mya (B phrase of Qingzang Movement) and climatic oscillation during middle Pleistocene (*c*. 0.64–0.36 Mya), rather than by the paleo-drainage systems. The present wide distribution of the species has resulted from recent population expansion (*c*. 0.053–0.025 Mya) from multiple refugia prior to the Last Glacial Maximum, corresponding to the scenario of “refugia within refugia”.

## Introduction

Genetic diversity and population structure of species are affected not only by their life histories and ecological traits [1], but also historical events [2], [3]. These mechanisms that shape genetic structure in natural populations might promote future diversification and preserve the evolutionary potential of extant species [4], and elucidating the factors that determine genetic structure of populations has been of longstanding interest to population geneticists [5].

The Yungui Plateau is adjacent to the Qinghai-Tibetan Plateau (QTP) and it is one of the world's biodiversity hotspots [6]. Accompanying the Late Cenozoic uplift of the QTP, which seems to have been the main driving force for shaping the current genetic structure and biodiversity of organisms in the region as revealed by previous studies (e.g. [7]), extremely complex topography and climate were formed in the Yungui Plateau. This significant increase in geological and ecological diversity promoted rapid divergence and speciation in small and isolated populations [7], and this region increasingly plays an important role in revealing biological consequences of the Late Cenozoic orogenic events [8].

Contemporary drainage systems develop very well on the Yunnan Plateau (e.g. Nu River, Lancang River, and Jinsha River). According to Clark et al. [9], modern upper and middle Yangtze River drainage and most of drainages distributed in Hengduan Mountains Region were originally major tributaries to the paleo-Red River system. These historical drainage rearrangements have been revealed as the main driving force for shaping the current genetic structure of *Nanorana yunnanensis* [10] and *Terminalia franchetii* [11], [12]. *Babina pleuraden* and *N. yunnanensis* have largely overlap distribution ranges [13]. The geological events that impacted *N. yunnanensis* may have the same impact on sympatric *B. pleuraden* and thus current genetic structure of *B. pleuraden* may also be associated with the paleo-drainage system.

Additionally, paleogeographical event is just one facet of the recent history of the earth, and Quaternary climatic oscillation has been proved to be equally important in shaping the genetic structure of many species. For regions that were glaciated and covered with continuous sheets of ice, species have been shown that they expanded from southern refugia after the Last Glacial Maximum (LGM) [3], [14]. China is one of the most important global Pleistocene refugia for lineages that evolved prior to the late Tertiary and Quaternary glaciations [15], but most of previous phylogeographic studies of impact caused by climatic changes mainly focused on taxa from QTP and adjacent mountain ranges (e.g. [16]) and southern China (e.g. [17]), and far less attention has been devoted to species endemic to southwestern China. In this region, the glaciations of the bordering montane regions appear to have occurred asynchronously relative to Northern Hemisphere glaciation events [18], and there was no unified ice-sheet covering the whole plateau throughout the Quaternary glaciations [19]. Thus, probably there were multiple refugia for species in southwestern China and their current distribution might result from asynchronous population expansion relative to expansion events of Northern Hemisphere species. We suppose that probably there were multiple refugia rather than single refugium for *B. pleuraden* during Quaternary climatic oscillation and its current distribution resulted from population expansion.

The Yunnan pond frog (*B. pleuraden*) is endemic to southwestern China, and its distribution covers the Yungui Plateau as well as southwestern Sichuan Basin [13]. This frog likes environments of standing water and it inhabits and breeds in montane paddy fields, ditches, and ponds from 1150–2300 m above sea level [13]. These make *B. pleuraden* an ideal model for investigating the effects of paleogeological (e.g. historical drainage rearrangements and uplift of plateau) and paleoclimatic events on species in southwestern China.

In this study, we use sequences of mitochondrial cytochrome *c* oxidase subunit I (COI) and cytochrome *b* (cyt *b*) to examine the phylogeographic pattern and genetic structure of *B. pleuraden*. Our specific objectives are to test for the following two hypotheses: i) Genetic structure of this standing-water dwelling frog is determined by the drainage history, and ii) There were multiple refugia rather than single refugium for *B. pleuraden* during Quaternary climatic oscillation and the species had experienced population expansion. Such information will not only shed light on the evolutionary history of this species, but also facilitate understanding of the historical and ongoing evolutionary driving forces for maintaining the extraordinarily high biodiversity of southwestern China.

## Methods

### Ethics statement

This study did not require any ethical or institutional approvals according to “Law of People's Republic of China on the Protection of Wildlife” and “Regulations for the Implementation of the People's Republic of China on the Protection of terrestrial Wildlife” because this species is not protected by any law and all sampling was conducted outside protected areas. In accordance with the requirements of “Regulation for the Collection of Genetic Resources (HJ 628–2011)”, only toe-clip tissues were collected and stored in 99% ethanol immediately after removal, and then animals were released immediately after treating wounds with antiseptic.

### Sample collection and laboratory procedures

**Figure 1:**
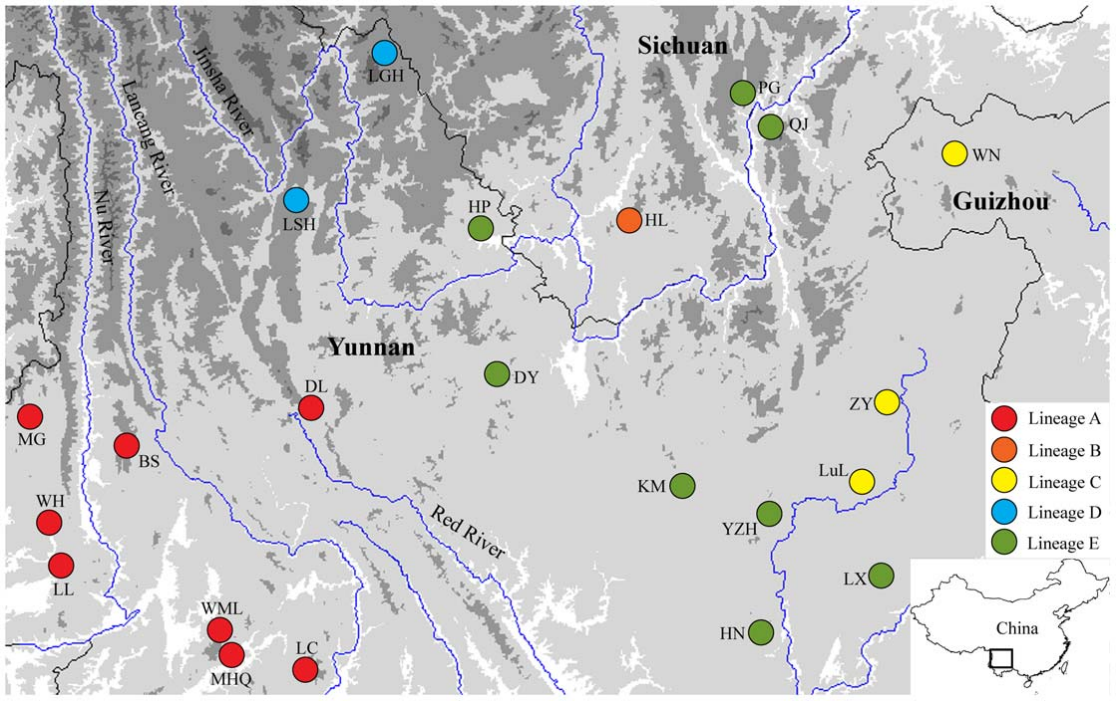
Map showing the sampled populations of *B. pleuraden*. Populations are named as in Table S1.

A total of 262 individuals were sampled from 22 populations throughout the entire geographical range of *B. pleuraden* (Table S1 and Figure 1). Two congeneric species, *Babina daunchina* and *Babina lini*, were selected as outgroups.

Tissue samples were digested using proteinase K, and genomic DNA was subsequently extracted following a standard phenol/chloroform isolation and ethanol precipitation. Fragments of cyt *b* and COI genes were amplified using primer pairs CBJ10933/Cyt*b*B [20] and L-turtCOIc/H-turtCOIc [21], respectively. PCR amplifications were performed in 50 µl reactions using the following cycling conditions: an initial denaturing step at 94°C for 3 min; 40 cycles of denaturing at 94°C for 60 s, annealing at 48°C for COI and at 53°C for cyt *b*, and one minute extension at 72°C, followed by a final extension step at 72°C for 10 min. PCR products were purified via spin columns. Sequencing was performed directly using the corresponding PCR primers. DNA sequences of both strands were obtained using the BigDye Terminator 3.1 on an ABI PRISM 3730 following the manufacture's instructions.

### Phylogenetic analyses

DNA sequences were aligned using CLUSTAL X 1.83 [22] with the default parameters and rechecked by eye. Haplotypes were identified using DnaSP 5.0 [23]. Prior to phylogenetic analyses, the degree of heterogeneity between cyt *b* and COI genes was investigated using the partition homogeneity test in PAUP* 4.0b10 [24] with 1000 replicates and 10 random sequence additions. The best-fit model of evolution was identified by Modeltest 3.7 [25]. Maximum likelihood (ML) and maximum parsimony (MP) analyses were performed using PAUP* 4.0b10. The MP method was performed using heuristic search with 1000 random-addition sequence replicates. ML analysis was performed using a heuristic search with 10 random-addition sequence replicates based on the best-fit substitution model. Support for nodes of the resulting MP and ML trees was assessed by analyses of 1000 bootstrap replicates in PAUP* 4.0b10 and 500 bootstrap replicates in PhyML 3.0 [26], respectively. Bayesian inference (BI) was performed using MrBayes 3.1.2 [27] based on the best-fit model of evolution, and two runs were performed simultaneously with four Markov chains starting from random trees. The Markov chains were run for 10^7^ generations and sampled every 100 generations. Convergence was confirmed by plots of Ln *L* scores and low standard deviation of split frequencies. The first 2×10^6^ generations were discarded as burn-in, and the remaining trees were used to create a 50% majority-rule consensus tree and to estimate Bayesian posterior probabilities (BPPs). Additionally, haplotype network was constructed using TCS 1.21 [28] based on the 95% parsimony criterion to infer the genealogical relationships among haplotypes.

### Estimating divergence times

Time to the most recent common ancestor (TMRCA) was estimated using an uncorrelated lognormal relaxed molecular clock model as implemented in Beast 1.6.2 [29]. To calibrate the rate of divergence, sequences of *Clinotarsus curtipes*, *Glandirana rugosa*, *Hylarana malabarica*, *Lithobates catesbeianus*, *Odorrana tormota*, *Odorrana ishikawae*, *Pelophylax chosenicus*, *Pelophylax kurtmuelleri*, *Pelophylax ridibundus*, *Rana chaochiaoensis* and *Staurois latopalmatus* (GenBank accession nos. shown in Table S2) were incorporated into the analysis. Due to the possible heterogeneity of evolutionary rates in different timescales [30], the analysis was performed in two steps following Teacher et al. [31]: the first using outgroups and a small number of divergent ingroup sequences and the second using no outgroup but all ingroup sequences. We included only five divergent haplotypes from the five clades (H7, H33, H35, H47 and H63; see results) in the dataset containing the outgroups. In this analysis, the divergence time between *P. kurtmuelleri* and *P. ridibundus* was set to 1.8 Mya [32] following a normal distribution and a minimum age of 1.4 Mya between *O. tormota* and *O. ishikawae* following a lognormal distribution based on the fossil records of *O. ishikawae* [33]. Three independent runs were conducted for 10^7^ generations using HKY+I+G model, assuming a Yule speciation process. The TMRCA of the five haplotypes was estimated from the three runs using Tracer v1.5 [34] after discarding the first 10^6^ generations as the burn-in. These estimates then were used as the calibration with a normal distribution for a second analysis, which included all samples but no outgroups. This analysis was performed for 10^7^ generations using HKY+G model, assuming a coalescent prior of constant population size. Mean rate was estimated in this analysis.

### Population analyses

Genetic diversity was estimated by haplotype diversity (*h*) and nucleotide diversity (*π*) in Arlequin 3.1 [35]. To test for the hypothesis that the pattern of population genetic structure of the species is determined by the drainage history, under which it is expected that geographical subdivision based on the paleo-river system will result in the highest value among group variation, hierarchical analysis of molecular variance (AMOVA) [36] was performed using Arlequin with 1000 permutations. This analysis identifies the optimal geographical subdivision for the data, by maximizing the among-group component (Φ_CT_) of the overall genetic variance. For this analysis, populations were grouped according to the mtDNA lineages recovered in phylogenetic analyses. Several other groupings were tested.

To test for the hypothesis that the species had experienced population expansion, under which a unimodal and approximately Poisson distributed mismatch distribution is expected, mismatch analysis was performed with Arlequin. Under the null hypothesis of sudden expansion, the raggedness index quantifying the smoothness of the observed mismatch distribution [37] and the sum of squared deviations (SSD) between observed and expected mismatch distribution were computed, and the statistical significance was tested by *P*_Rag_ and *P*_SSD_, respectively, using a bootstrap approach (1000 replicates). The time at which the population expansion began (*t*) can be estimated from the relationship τ = 2*ut* [38], where τ is the mode of the mismatch distribution and *u* is the estimated mutation rate of the sequences. Additionally, the hypothesis of population expansion was also tested using Fu's *F*_S_ test [39] and Tajima's *D* statistics [40], both of which are expected to have significant negative values under demographic expansion. Both statistics were calculated with Arlequin and their significance was assessed through 1000 simulations.

### Tests of glacial refugia assumptions based on coalescent simulations

Coalescent simulations of genealogies constrained within models of population divergence provide a powerful means of assessing the fit of observed genetic patterns to different phylogeographic hypotheses [41]. To test for the hypothesis that there were multiple glacial refugia rather than single refugium for this species during Quaternary climatic oscillation, 1000 coalescent genealogies were generated in Mesquite 2.74 [42] under each historical scenario, and the distribution of *S*, the minimum number of sorting events required to explain the population subdivision [43], was recorded. Then, we evaluated model fit by comparing the *S*-value of ML genealogy and the *S*-values of the simulated genealogies.

Effective population size (*N*e) for the simulations was estimated using the mutation parameter *θ* (*Theta-W*) calculated by DnaSP. We converted the theta to *N*e using the equation *θ* = 2*N*eμ, with the estimated mean substitution rate. During coalescent simulations, the overall *N*e was set to equal the empirical estimate, and the *N*e of the refugia population was constrained to a size proportional to the overall *N*e. Absolute time (years) was converted to coalescent time (generations), assuming a generation time of 2.5 years. TMRCA dates estimated from Beast were used to construct multiple refugia hypothesis (detailed in Results).

## Results

### Sequence characteristics

The aligned sequences of cyt *b* and COI fragments were 525 bp and 723 bp in length, respectively. From 262 individuals sequenced, we obtained 43 haplotypes of cyt *b* and 37 haplotypes of COI that were defined by 109 and 117 polymorphic sites, respectively. No insertions or deletions were observed. A total of 63 haplotypes (H1–H63) were defined by 226 polymorphic sites when cyt *b* and COI sequences were combined. Of the 63 haplotypes, only six (H10, H26, H36, H40, H50, H58) were shared among different sampling sites from same regional group and no haplotype was shared among different regional groups (Table S1). The sequences of 65 cyt *b* and COI haplotypes (including two outgroups) have been submitted to GenBank: accession numbers HQ395289–HQ395353.

### Phylogeny and divergence time

The partition homogeneity test revealed no significant conflicting phylogenetic signals between cyt *b* and COI genes (*P* = 0.087), so all further analyses was performed on the combined data. The HKY+G model was selected as the best-fit model of nucleotide substitution.

**Figure 2:**
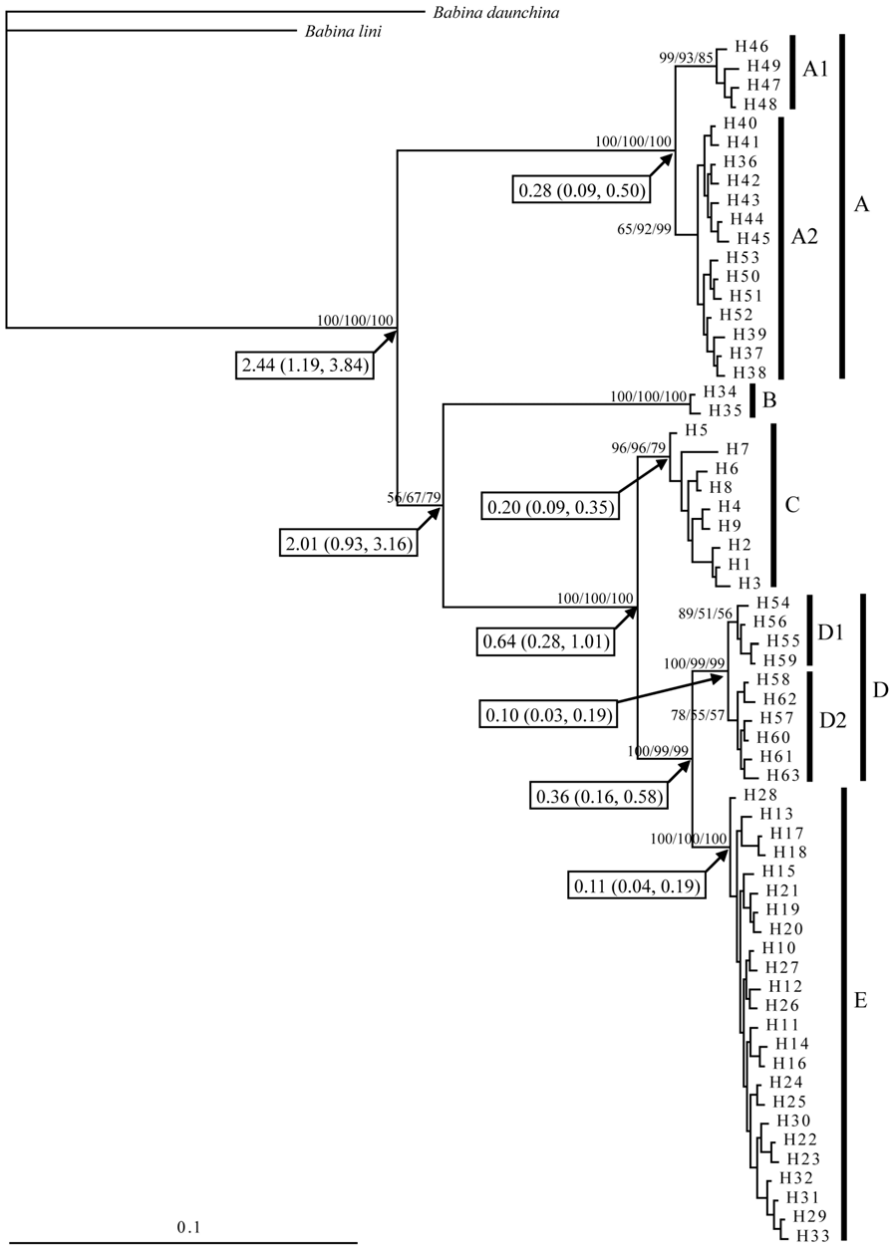
Bayesian inference tree for *B. pleuraden* based on the 63 haplotypes from cyt *b* and COI sequences. The nodal numbers are BPP, ML, and MP bootstrap values, respectively. Estimated dates in Mya with 95% HPD are given in rectangular boxes.

ML, MP, and BI analyses produced highly consistent tree topologies (Figure 2). Monophyly of the 63 haplotypes was strongly supported and five distinctive lineages (A–E) were recognized, with two of these (A and D) being further subdivided into two smaller clades (A1 and A2, D1 and D2). Lineage A was comprised of haplotypes from the western Yunnan Plateau, and A1 haplotypes occurred only in MG. Haplotypes of lineage B (H34–H35) were restricted to HL in the southwestern Sichuan Basin, and haplotypes (H1–H9) in lineage C were confined to the three populations (WN, ZY, and LuL) from the eastern Yunnan Plateau. Haplotypes in lineage D (H54–H63) were confined to LSH and LGH in the northwestern Yunnan Plateau, and D1 only comprised haplotypes from LSH whereas D2 comprised haplotypes from both LSH and LGH. Haplotypes from the central Yunnan Plateau were included in lineage E. In all cases, lineages D and E were recovered as the sister group to each other, and then lineage C was the sister group to lineage D plus lineage E with strong support values. Lineage B was recovered as the sister group to the clade (C, (D, E)), although only maximum parsimony analysis given strong support value.

The separation time of lineage A from all other lineages was estimated to be *c*. 2.44 (95% HPD: 1.19–3.84) Mya. Subsequently, lineage B separated from lineages C, D, and E approximately 2.01 (95% HPD: 0.93–3.16) Mya, lineage C separated from lineages D and E approximately 0.64 (95% HPD: 0.28–1.01) Mya, and lineage D separated from lineage E approximately 0.36 (95% HPD: 0.16–0.58) Mya (Figure 2).

### Population structure

Hierarchical analysis of molecular variance (AMOVA) revealed that grouping based on the five major lineages (A–E), which roughly corresponds to the five geographical regions ([western Yunnan Plateau], [southwestern Sichuan Basin], [eastern Yunnan Plateau], [northwestern Yunnan Plateau], [central Yunnan Plateau]), respectively, resulted in the highest value among group variation (Φ_CT_ = 0.95141, *P*<0.001) and was inferred to be the most probable geographical subdivision (Table S3). Site of lineage B is surrounded by sites of lineage E, this might have been contributed by population expansion of lineage E (see below). A long-term interruption of gene flow among lineages was evidenced by the high Φ_ST_ values (Table S3).

**Figure 3:**
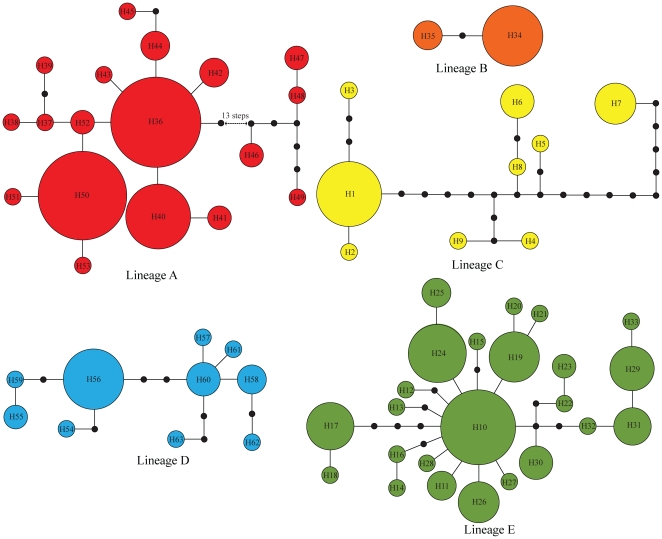
Networks of the 63 haplotypes detected from cyt *b* and COI sequences of *B. pleuraden*. The sizes of the circles are proportional to haplotype frequencies and black dots represent missing haplotypes (not sampled or extinct).

TCS analysis obtained five haplotype networks at 95% probability (connection limit = 15), corresponding to the five major lineages obtained by phylogenetic analyses (Figure 3). No alternative connections between haplotypes (‘loops’) were observed, indicating that no homoplasy was involved in the network.

### Glacial refugia testing

**Figure 4:**
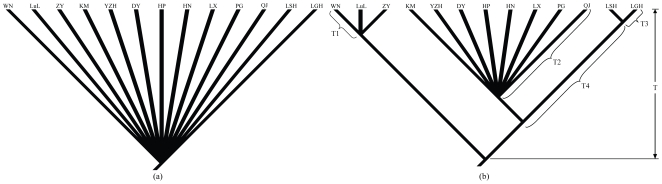
Models used to test Pleistocene refugial hypotheses. (a) Single refugium hypothesis: populations from eastern, northwestern, and central Yunnan Plateau are derived from a single refugium and began to expand at the end of the last glacial maximum (*c*. 18 000 years BP). (b) Multiple refugia hypothesis: two lineages split at the Middle Pleistocene (T = 640 000 years BP), with the northwestern plus central branch diverged into two clades at 360 000 years ago (T3+T4). T1 (200 000 years BP), T2 (110 000 years BP), and T3 (100 000 years BP) were derived from the estimates of TMRCA.

Based on the empirical *θ* values and the estimated mean rate of 0.036 substitutions per site per million years, the effective population size was calculated (Table 1). The following two hypotheses concerning the glacial refugia were tested. The first was a single refugium hypothesis that extant populations from the northwestern, central, and eastern Yunnan Plateau derived from a single refugium at the end of the LGM (*c*. 0.018 Mya; Figure 4a). The second was a multiple refugia hypothesis that populations from the eastern Yunnan Plateau (lineage C) were isolated from populations from northwestern and central Yunnan Plateau at middle Pleistocene (*c*. 0.64 Mya), with populations from northwestern and central Yunnan Plateau diverging into two further lineages (D and E) (*c*. 0.36 Mya; Figure 4b). The basis of the second hypothesis is that populations from the northwestern, central, and eastern Yunnan Plateau form their own clades, respectively.

**Table 1:** Estimation of the empirical theta values and effective population size.

Lineage	*Theta-W*	*N*e
Total (C+D+E)	0.01361	75611
C	0.00582	32333
D	0.00268	14889
E	0.00503	27944

**Figure 5:**
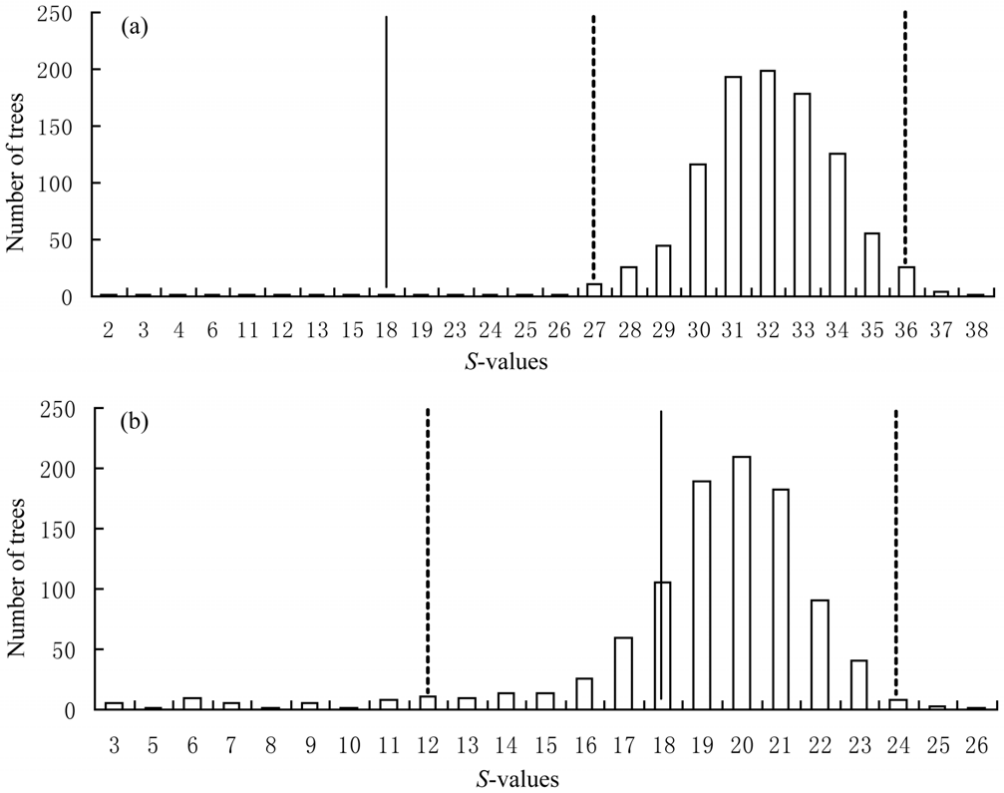
*S*-values for 1000 simulated coalescent genealogies. (a) Results from simulations within the single refugium hypothesis; (b) Results from simulations within the multiple refugia hypothesis. The black line represents the *S*-value for our ML genealogy and the dashed lines represent the 95% CI for the distribution.

We calculated *S* = 18 for our ML genealogy. This value does not falls within the 95% confidence interval of the simulated distribution of *S* for the single refugium hypothesis, but falls within the 95% confidence interval of the simulated distribution of *S* for the multiple refugia hypothesis (Figure 5). So we reject the hypothesis of single refugium in favor of the multiple refugia hypothesis.

### Demographic history

**Figure 6:**
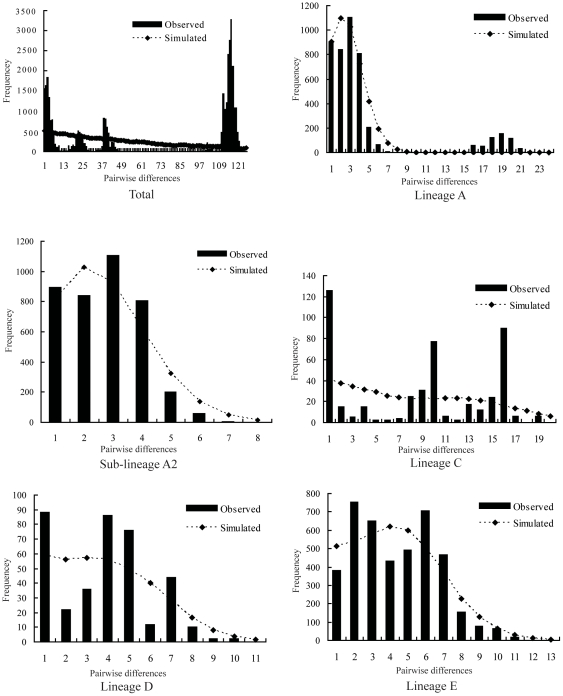
Mismatch distributions for total samples and some major lineages of *B. pleuraden*.

Unimodal curve, indicative of demographic expansion, was observed for both lineages D and E (Figure 6), and the variance (SSD) and raggedness index suggested that the curves did not significantly differ from the distributions expected from a model of sudden population expansion (*P*>0.05, Table 2). Although lineage A had multimodal curve, A2 had unimodal curve with non-significant values of *P*_SSD_ and *P*_Rag_ (*P*>0.05) and the value of *F*_S_ was significantly negative (*P*<0.05), supporting the population expansion model within sub-lineage A2. Significantly negative values of *F*_S_ and Tajima's *D* statistics were obtained for lineage E, also supporting that this lineage had undergone a sudden demographic expansion. For lineage C, both mismatch analysis and neutrality tests rejected the sudden demographic expansion hypothesis. Based on the estimated mean rate of 0.036 substitutions per site per million years, we calculated the demographic expansion of A2, D, and E to have occurred *c*. 0.025, 0.049, and 0.053 Mya, respectively (Table 2).

**Table 2:** Results of mismatch distribution analysis, neutrality test, and estimation of expansion time.

			Raggedness	Expansion		
Lineage	τ	SSD (*P*_SSD_)	index (*P*_Rag_)	time (Mya)	Fu's *F_S_* (*P*-value)	Tajima's *D* (*P*-value)
A	2.383	0.00967 (0.336)	0.02831 (0.826)	–	−3.05698 (0.144)	−1.36235 (0.053)
A2	2.209	0.00822 (0.363)	0.03593 (0.722)	0.025	−5.20566 (0.019)	−0.77987 (0.254)
C	14.371	0.09234 (0.028)	0.14919 (0.012)	–	3.24223 (0.881)	0.19585 (0.636)
D	4.375	0.03569 (0.239)	0.09445 (0.213)	0.049	−1.82020 (0.192)	−0.47442 (0.361)
E	4.738	0.01051 (0.467)	0.02343 (0.671)	0.053	−9.36401 (0.003)	−1.43826 (0.049)
Total	0.000	0.03699 (0.063)	0.00663 (0.018)	–	23.91200 (0.976)	2.67032 (0.996)

## Discussion

### Genetic structure and uplift of the QTP

The results of this study showed five main mitochondrial haplotype lineages (A–E) within *B. pleuraden* (Figure 2 and Figure 3), roughly corresponding to five regional groups. Lineage A includes all haplotypes from the western Yunnan Plateau and it is basal to all other lineages. This pattern is not compatible with the paleo-drainage history that the modern upper and middle Yangtze River drainage and most of drainages distributed in Hengduan Mountains Region were originally major tributaries to the paleo-Red River system [9], under which the western lineage would cluster together with the central lineage as revealed in the phylogeographic study of *N. yunnanensis* [10]. This is also evidenced by the AMOVA analysis, which revealed that the greatest amount of genetic variation occurred among populations within groups (76.85%) when the data were partitioned into two groups as eastern lineage (C) vs. western plus central lineages (A, B, D, E), whereas the greatest amount of genetic variation occurred among groups (78.06%) when the data were partitioned into two groups as western lineage (A) vs. central plus eastern lineages (B, C, D, E) (Table S3). This incongruence indicates that unlike *N. yunnanensis*, *B. pleuraden* had experienced different evolutionary history and its genetic structure was determined by other factors rather than the paleo-river system.

Genetic diversity and population structure of species can be affected by their life histories and ecological traits [1]. A possible factor leading to this inconsistence between *N. yunnanensis* and *B. pleuraden* is habitat preference. *Nanorana yunnanensis* is associated with lotic environments [13], flowing water may significantly contribute to the gene flow among populations from same river system [44] through movement of tadpoles and eggs [45], and consequently its genetic structure is dominated by the paleo-river system. Conversely, *B. pleuraden* lives and breeds in lentic environments such as paddy fields, ditches, and ponds [13], and standing water bodies can act as barriers to gene flow among populations [46] and probably large geologic events and processes representing longer time scales might overshadow the structuring effects of drainages [47].

Although geographically the Red River Fault locates between sites of lineage A and that of other lineages, it seems to have no contribution to the split between western and other lineages because its right-lateral slip started from 4.7 Mya [48], and the divergence time between western and other lineages was dated to be *c*. 2.44 Mya. This divergence event is consistent with the disruption of planation surface in the Yunnan Plateau occurred early Pleistocene [8]. During Pliocene, the Yunnan Plateau was under the setting of weak tectonic movement [8], [49]. However, strong tectonic movement in the Yunnan Plateau was caused by the accelerated uplift of the QTP that started from 2.5 Mya (phase B of Qingzang Movement [50], [51]), to which different regions of the Yunnan Plateau responded uniquely and the affecting strength gradually weakened from west to east, and the sinking speeds of basins in the western region were faster than that of basins in the central and eastern regions [8]. As a consequence of regional responses to the phase B of Qingzang Movement, punctiform-retiform drainage network was disrupted and fluvial system was formed in the western region during early Pleistocene, whereas punctiform-retiform drainage network was kept in other regions [8]. This change might have contributed to the interruption of gene flow between western and other lineages because of this species' preference of lentic environment.

According to Clark et al. [9], middle Jinsha River drainage originally flowed southward as tributary to the paleo-Red River. So populations in the southwestern Sichuan Basin and central regions of the Yunnan Plateau would cluster together. This pattern has been observed in *N. yunnanensis* [10] and *T. franchetii*, a plant endemic to the river valleys of Southwest China [11], [12]. However, in the present study, haplotypes from HL formed a distinctive lineage (B; Figure 2 and Figure 3) and it diverged from lineages C, D, and E approximately 2.01 Mya (Figure 2). AMOVA analysis revealed that Φ_CT_ values were obviously lower when lineage B was grouped together with lineages D and/or E (Table S3). These results do not reflect above history of paleo-river system and further indicate that genetic structure of *B. pleuraden* was not determined by the paleo-river system. It might represent a relic population owing to the rapid and extreme uplift of the QTP, which lead to the significantly increase in geological and ecological diversity of the QTP and its adjacent regions as well as rapid divergence and speciation in small and isolated populations [7], and consequently confined these haplotypes at southwestern Sichuan Basin accompanied with long-term interruption of gene flow between HL and other populations. A previous phylogeographic study on species level has also found that species from the southwestern Sichuan Basin formed a distinct clade that diverged from other clades during the early Pleistocene [52]. Current geographical boundary between lineages B and E is not very clear, which might have been shaped by the past population expansion of lineage E (see below).

### Genetic structure and Quaternary climatic oscillations

Although the Xiaojiang Fault located between sites of lineage C and that of other lineages, it seems to have no contribution to the divergence of lineage C because its left-lateral slip started from 2–4 Mya [53]. It was known that the dramatic climatic oscillations of the Quaternary period had a profound effect on the current distribution and genetic structure for most living organisms [14]. During late and middle Pleistocene, three to five glaciations occurred in the montane regions of western China, but no continuous ice sheet was present [54] and environmental diversity of tropics and subtropics in lower elevation were still maintained [55]. In the present study, lineage C diverged from lineages D and E approximately 0.64 Mya, and the split between lineages D and E occurred *c*. 0.36 Mya (Figure 2). These two divergence events coincide with the Naynayxungla glaciation at 0.78–0.5 Mya and the first stage of the Penultimate glaciation at 0.32±0.06 Mya [56], respectively. The species might have survived at three refugia on the northwestern, central, and eastern Yunnan Plateau during the Quaternary climatic oscillations. This is evidenced by our coalescent simulations, which reject the hypothesis that populations from the northwestern, central, and eastern Yunnan Plateau derived from a single refugium in favor of the hypothesis of multiple refugia. Indeed, molecular evidence for refugial survival on the northwestern and eastern Yunnan Plateau has also been recently reported by Zhan et al. [57] and Wang et al. [58], respectively. The current wide distribution of *B. pleuraden* can be attributable to the population expansion during interglacial period, which is supported by demographic analyses (see below). With repeated range changes, surviving populations may pass through many adaptations and reorganizations, allowing their lineages to diverge and accumulate genetic differences [3], [14]. Within lineage E, KM has the highest haplotype diversity, a large number of private haplotypes, and the ancestral haplotype (H10) is present, suggesting that this area has serviced as the main refugium for this lineage. Similarly, ZY might have serviced as the main refugium for lineage C.

Several recent studies supported southwestern China as a major glaciation refugium (e.g. [59]). Multiple refugia in southwestern China for *B. pleuraden* revealed here is consistent with the scenario of “refugia within refugia” described by Gómez and Lunt [60]

### Demographic history

Quaternary climatic changes have shaped patterns of geographical distribution as well as demographic history [5]. Existence of multiple glacial refugia supported by the coalescent simulations implies that the current distribution of *B. pleuraden* in northwestern, central and eastern Yunnan Plateau might have resulted from multiple independent expansion events. In addition, co-occurrence of A1 and A2 and higher nucleotide diversity (0.008324±0.004761) in MG suggest secondary contact and so population expansion. These expectations are supported by the mismatch analysis, which detected sign of population expansion within lineages A, D, and E with the exception of C. Contrary to the mismatch analysis, neutrality tests revealed no strong sign of demographic growth within lineages A and D (Table 2). This incongruence could be attributed to the occurrence of subdivisions within them because mismatch analysis is robust and hardly affected by population structure [37] and should approximately hold true even when populations are completely isolated [61], whereas population subdivision lowers the power of neutrality tests [62]. In fact, sign of population expansion was detected in sub-lineage A2 by both mismatch analysis and Fu' *F*_S_ test (Table 2).

The estimation of expansion time for *B. pleuraden* (*c*. 0.053–0.025 Mya, Table 2) is prior to the Last Glacial Maximum (LGM, 0.023–0.018 Mya), after which many European and North American species expanded from southern refugia [3], [14]. This is consistent with the hypothesis that glaciations of the bordering montane regions of southwestern China appear to have occurred asynchronously relative to Northern Hemisphere glaciation events [18], and there was no uniform ice sheet [19], [54]. Most recent studies of species from western China also obtained an estimation of expansion time prior to the LGM (e.g. [10], [16], [57]).

According to Hewitt [14], populations likely rapidly colonized or recolonized some areas during climatic oscillations, which would reduce allelic diversity within lineages. This is reflected well by the fact that higher level of haplotype diversity and lower level of nucleotide diversity occurred in lineages (A, D, and E) undergoing historical demographic expansion, whereas lower level of haplotype diversity and higher level of nucleotide diversity were observed in lineage (C) exhibiting demographic stability (Table S1). The DL population was fixed for H36, indicating a rapid process of population colonization eastward to DL accompanied with strong founder effect.

### Implication for conservation

*Babina pleuraden* was listed as a species of least concern by IUCN [63], but there was a shrinking trend in the distribution of this species compared to its historical records [64], owing to threats such as habitat destruction and degradation caused by urbanization and water pollution, but also to over-harvesting by local people [63]. In the present study, we find that *B. pleuraden* comprises five regional lineages and significant population differentiation, indicating strong population genetic structure and high genetic diversity. These five regional lineages may represent important components in the evolutionary and adaptive structure of this species, and thus future conservation policy for this species should concentrate on protection of these distinct lineages and maintenance of genetic diversity because goals of any conservation program should be to ensure the survival of a species and preserve its genetic diversity for long-term evolutionary success [65].

### Conclusions

In summary, our results indicate that *B. pleuraden* comprises five well-supported mitochondrial lineages and its phylogeographic pattern has been shaped by the unique regional responses of the Yunnan Plateau to the rapid uplift of the Qinghai-Tibetan Plateau during early Pleistocene and climatic oscillation during middle Pleistocene, rather than by the paleo-drainage systems. The present wide distribution of this species has resulted from population expansion from multiple refugia prior to the Last Glacial Maximum. Considering that this study is completely based on mitochondrial data, results presented here should be treated with caution.

## Supporting Information

Table S1 **Sampling localities, phylogroup, sample sizes (** ***N*** **), mtDNA haplotypes and their frequencies, as well as estimates of gene diversity and nucleotide diversity.**
(DOC)

Table S2 **Species and sequences incorporated into the analysis of divergence dating.**
(DOC)

Table S3 **Results of hierarchical analysis of molecular variance (AMOVA).**
(DOC)
