# Supplementary material for: Phylogeography and Demographic History of Babina pleuraden (Anura, Ranidae) in Southwestern China
Source: PLoS One. 2012 Mar 20;7(3):e34013. doi: 10.1371/journal.pone.0034013 (PMC3309021; doi:10.1371/journal.pone.0034013)
Supplement: Table S1 — Sampling localities, phylogroup, sample sizes ( N ), mtDNA haplotypes and their frequencies, as well as estimates of gene diversity and nucleotide diversity. (DOC) [file pone.0034013.s001.doc]

**Table S1** Sampling localities, phylogroup, sample sizes (*N*), mtDNA haplotypes and their frequencies, as well as estimates of gene diversity and nucleotide diversity.

| Lineage | Population | °N Latitude | °E Longitude | Haplotypes present (number of individuals) | *N* | Haplotype diversity | Nucleotide diversity |
| --- | --- | --- | --- | --- | --- | --- | --- |
| C |  |  |  |  |  | 0.7290 ± 0.0704 | 0.006135 ± 0.003270 |
|  | Weining (WN) | 26°51′26″ | 104°16′28″ | H1 (15), H2 (1), H3 (1) | 17 | 0.2279 ±0.1295 | 0.000377 ± 0.000386 |
|  | Luliang (LuL) | 25°01′46″ | 103°39′59″ | H4 (1) | 1 | – | – |
|  | Zhanyi (ZY) | 25°36′01″ | 103°49′19″ | H5 (1), H6 (4), H7 (6), H8 (1), H9 (1) | 13 | 0.7308 ±0.0963 | 0.006513 ± 0.003634 |
| E |  |  |  |  |  | 0.9095 ± 0.0155 | 0.002692 ± 0.001543 |
|  | Kunming (KM) | 25°07′48″ | 102°26′32″ | H10 (5), H11 (3), H12 (1), H13 (1), H14 (1), H15 (1), H16 (1) | 13 | 0.8333 ± 0.0861 | 0.001623 ± 0.001095 |
|  | Yangzonghai (YZH) | 24°54′33″ | 102°59′20″ | H17 (8), H18 (1) | 9 | 0.2222 ± 0.1662 | 0.000178 ± 0.000262 |
|  | Dayao (DY) | 25°51′35″ | 101°13′47″ | H19 (9), H20 (1), H21 (1) | 11 | 0.3455 ± 0.1722 | 0.000291 ± 0.000341 |
|  | Huaping (HP) | 26°37′49″ | 101°15′55″ | H22 (1), H23 (2) | 3 | 0.6667 ± 0.3143 | 0.000534 ± 0.000666 |
|  | Huaning (HN) | 24°11′36″ | 102°55′42″ | H10 (2), H24 (12), H25 (3) | 17 | 0.4853 ± 0.1260 | 0.000424 ± 0.000416 |
|  | Luxi (LX) | 24°31′38″ | 103°45′50″ | H10 (13), H26 (1), H27 (1), H28 (1) | 16 | 0.3500 ± 0.1478 | 0.000300 ± 0.000338 |
|  | Puge (PG) | 27°22′28″ | 102°32′25″ | H29 (7), H30 (4), H31 (5), H32 (1), H33 (1) | 18 | 0.7582 ± 0.0601 | 0.001728 ± 0.001124 |
|  | Qiaojia (QJ) | 27°13′57″ | 103°02′36″ | H26 (5) | 5 | 0.0000 ± 0.0000 | 0.000000 ± 0.000000 |
| B |  |  |  |  |  | 0.3250 ± 0.1251 | 0.000521 ± 0.000476 |
|  | Huili (HL) | 26°39′20″ | 102°14′40″ | H34 (13), H35 (3) | 16 | 0.3250 ± 0.1251 | 0.000521 ± 0.000476 |
| A |  |  |  |  |  | 0.7987 ± 0.0250 | 0.002884 ± 0.001635 |
|  | Dali (DL) | 25°36′25″ | 100°16′00″ | H36 (14) | 14 | 0.0000 ± 0.0000 | 0.000000 ± 0.000000 |
|  | Lingcang (LC) | 23°57′20″ | 100°15′32″ | H37 (1), H38 (1), H39 (1) | 3 | 1.0000 ± 0.2722 | 0.001603 ± 0.001511 |
|  | Wumulong (WML) | 24°12′03″ | 99°42′22″ | H40 (6), H41 (2) | 8 | 0.4286 ± 0.1687 | 0.000343 ± 0.000391 |
|  | Mahuangqin (MHQ) | 24°06′15″ | 99°44′32″ | H36 (2), H40 (9), H42 (3) | 14 | 0.5604 ± 0.1245 | 0.000687 ± 0.000578 |
|  | Baoshan (BS) | 25°07′41″ | 98°43′48″ | H36 (9), H43 (1), H44 (3), H45 (1) | 14 | 0.5714 ± 0.1322 | 0.000696 ± 0.000583 |
|  | Mingguang (MG) | 25°29′29″ | 98°32′31″ | H46 (2), H47 (2), H48 (1), H49 (1), H50 (3) | 9 | 0.8611 ± 0.0872 | 0.008324 ± 0.004761 |
|  | Wuhe (WH) | 24°51′59″ | 98°40′11″ | H50 (11), H51 (1) | 12 | 0.1667 ± 0.1343 | 0.000134 ± 0.000217 |
|  | Longling (LL) | 24°36′14″ | 98°41′38″ | H36 (4), H50 (14), H52 (2), H53 (1) | 21 | 0.5333 ± 0.1114 | 0.000679 ± 0.000559 |
| **Table S1** (*continued*) | | | | | | | |
| Lineage | Population | °N Latitude | °E Longitude | Haplotypes present (number of individuals) | *N* | Haplotype diversity | Nucleotide diversity |
| D |  |  |  |  |  | 0.7672 ± 0.0750 | 0.002298 ± 0.001387 |
|  | Lashihai (LSH) | 26°52′21″ | 100°07′24″ | H54 (1), H55 (2), H56 (13), H57 (1), H58 (1), H59 (1) | 19 | 0.5380 ± 0.1330 | 0.001415 ± 0.000958 |
|  | Luguhu (LGH) | 27°39′41″ | 100°46′54″ | H58 (2), H60 (4), H61 (1), H62 (1), H63 (1) | 9 | 0.8056 ±0.1196 | 0.001469 ± 0.001054 |
| Total |  |  |  |  | 262 | 0.9540 ± 0.0048 | 0.054781 ± 0.026206 |
