# Supplementary material for: Phylogeography and Demographic History of Babina pleuraden (Anura, Ranidae) in Southwestern China
Source: PLoS One. 2012 Mar 20;7(3):e34013. doi: 10.1371/journal.pone.0034013 (PMC3309021; doi:10.1371/journal.pone.0034013)
Supplement: Table S2 — Species and sequences incorporated into the analysis of divergence dating. (DOC) [file pone.0034013.s002.doc]

**Table S2** Species and sequences incorporated into the analysis of divergence dating.

| Species | GenBank accession no. | |
| --- | --- | --- |
|  | COI | Cyt *b* |
| *Clinotarsus curtipes* | GU136127 | AF249079 |
| *Glandirana rugosa* | AB511301 | AF205092 |
| *Hylarana malabarica* | GU136126 | GU136140 |
| *Lithobates catesbeianus* | EF525854 | AF205089 |
| *Odorrana tormota* | DQ835616 | DQ835616 |
| *Odorrana ishikawae* | NC015305 | NC015305 |
| *Pelophylax chosenicus* | NC016059 | NC016059 |
| *Pelophylax kurtmuelleri* | JN871599 | ‒ |
| *Pelophylax ridibundus* | JN700830 | AB640977 |
| *Rana chaochiaoensis* | JF939103 | JF939140 |
| *Staurois latopalmatus* | AB511311 | ‒ |
