# Supplementary material for: Phylogeography and Demographic History of Babina pleuraden (Anura, Ranidae) in Southwestern China
Source: PLoS One. 2012 Mar 20;7(3):e34013. doi: 10.1371/journal.pone.0034013 (PMC3309021; doi:10.1371/journal.pone.0034013)
Supplement: Table S3 — Results of hierarchical analysis of molecular variance (AMOVA). (DOC) [file pone.0034013.s003.doc]

**Table S3** Results of hierarchical analysis of molecular variance (amova).

| Grouping option | Φ_ST_ | Φ_SC_ | Φ_CT_ | Among groups | Among populations within groups | Within populations |
| --- | --- | --- | --- | --- | --- | --- |
| 1 group [A, B, C, D, E] | 0.97832** |  |  |  | 97.83% | 2.17% |
| 2 groups [A]; [B, C, D, E] | 0.98697** | 0.94061** | 0.78059** | 78.06% | 20.64% | 1.30% |
| 2 groups [A, C, D, E]; [B] | 0.98633** | 0.97642** | 0.42028* | 42.03% | 56.61% | 1.37% |
| 2 groups [A, B, D, E]; [C] | 0.98193** | 0.97702** | 0.21345 | 21.34% | 76.85% | 1.81% |
| 3 groups [A]; [B]; [C, E, D] | 0.98658** | 0.89452** | 0.87278** | 87.28% | 11.38% | 1.34% |
| 3 groups [A]; [B, D, E]; [C] | 0.98484** | 0.92812** | 0.78909** | 78.91% | 19.58% | 1.52% |
| 4 groups [A]; [B, E]; [C]; [D] | 0.98329** | 0.91802** | 0.79616** | 79.62% | 18.71% | 1.67% |
| 4 groups [C]; [E]; [B, D]; [A] | 0.98294** | 0.89880** | 0.83144** | 83.14% | 15.15% | 1.71% |
| 4 groups [A]; [B]; [C]; [D, E] | 0.98472** | 0.81539** | 0.91724** | 91.72% | 6.75% | 1.53% |
| 5 groups [A ]; [B]; [C]; [D]; [E] | 0.98332** | 0.65677** | **0.95141**** | 95.14% | 3.19% | 1.67% |

* *P* < 0.05; ** *P* < 0.001
